# Improving police recorded crime data for domestic violence and abuse through natural language processing

**DOI:** 10.3389/fsoc.2025.1686632

**Published:** 2025-11-24

**Authors:** Darren Cook, Ruth Weir, Leslie Humphreys

**Affiliations:** 1 Violence and Society Centre, City University of London, London, United Kingdom; 2 School of Law and Policing, University of Lancashire, Preston, United Kingdom

**Keywords:** natural language processing, police recorded crime, domestic violence (DV), text classication, supervised machine learning, DistilBERT, free text

## Abstract

**Introduction:**

Domestic Violence and Abuse (DVA) is a growing public health and safeguarding concern in the UK, compounded by long-standing data quality issues in police records. Incomplete or inaccurate recording of key variables undermines the ability of police, health services, and partner agencies to assess risk, allocate resources, and design effective interventions.

**Methods:**

We evaluated two machine learning models (Random Forest and DistilBERT) for classifying the type of victim/offender relationship (ex-partner, current partner, and family) from approximately 19,000 DVA incidents recorded by a UK police force. Models were benchmarked against a static rule-based classifier and assessed using precision, recall, and F1-score. To reduce false positives in the most challenging relationship categories, we implemented a selective classification strategy that abstained from low-confidence predictions.

**Results:**

Both machine learning models outperformed the baseline across all metrics, with average absolute gains of 11% in precision and 16% in recall. Ex-partner cases were classified most accurately, while current partner cases were classified with the least accuracy. Selective classification substantially improved precision for underperforming categories, albeit at the expense of reduced coverage.

**Discussion:**

These findings demonstrate that computational tools can enhance the completeness and reliability of police DVA data, provided their use balances predictive accuracy, interpretability, and safeguarding risks.

## Introduction

Domestic Violence and Abuse (DVA) is a significant and growing problem in the UK. In England and Wales, over 2.3 million people aged 16 and over are estimated to experience DVA every year, and police record more than 1.3 million DVA incidents per year (Office for National Statistics, 2024). DVA disproportionately affects women (Barrow-Grint et al., 2022; HMICFRS, 2019), with 6.6% of women aged 16 and over experiencing abuse in the 12 months to March 2024 (Office for National Statistics, 2024).

The statutory definition of DVA in England and Wales includes those who are “personally connected,” meaning both intimate partners and relatives fall within the definition (Domestic Abuse Act, 2021). Previous research has found that risk factors for repeat victimization vary according to the type of relationship, with partners and ex-partners exhibiting more similar risk profiles compared to other familial relationships (Weir, 2024). The emotional and physical impacts of abuse have also been found to vary by relationship type (Blom et al., 2024), highlighting the importance of police accurately and consistently recording this field. In its 2017 progress report on police responses to DVA, Her Majesty's Inspectorate of Constabulary, Fire and Rescue Services (HMICFRS) included in its checklist of data for scrutiny by Police and Crime Commissioners (PCCs), whether forces record DVA incidents broken down by the relationship between victim and perpetrator (HMICFRS, 2015).

Accurate police-recorded crime data is vital for understanding and responding to DVA, yet it often contains missing values and inaccuracies. Across all crime types, the quality of police data in England and Wales has long been a concern. In 2014, weaknesses in data collection and processing led to police-recorded crime statistics losing their National Statistics accreditation. While there have been improvements in overall crime data recording since then (Whitehead, 2024), individual police forces still encounter difficulties adequately recording instances of DVA in police-recorded crime datasets (HMICFRS, 2019). To our knowledge, there has been no detailed analysis of the completeness or accuracy of specific data fields within these records.

Poor recording practices undermine investigations and the overall response to DVA, which increases the risk of harm to victims (Phoenix, 2023). Incomplete or inconsistent data makes it harder to identify strategic trends, detect escalation in abusive behavior, and carry out accurate risk assessments. As a result, police may miss opportunities to intervene early, fail to connect related incidents, and misjudge the level of danger victims face (Myhill and Hohl, 2019).

Correcting poorly recorded or missing data at this scale is non-trivial and beyond the capabilities of manual intervention alone. Fortunately, the increasing availability of computational solutions and machine learning algorithms can augment, and to a degree, offset much of this processing (Neubauer et al., 2023). The application of text mining and natural language processing (NLP) solutions across industries is supported by a growing body of interdisciplinary research, which shows that valuable information can be automatically extracted from unstructured data such as crime reports and case summaries through technology (Karystianis et al., 2019; Victor et al., 2021).

However, automated prediction systems are not without risk, particularly when applied in sensitive domains such as policing. Data inherently reflects societal biases that poorly designed AI solutions can amplify (Leitgöb et al., 2023; Lum and Isaac, 2016). In the context of DVA, these biases may stem from underreporting of marginalized demographic groups or inconsistencies in police recording practices. Left unaddressed, predictive systems could misclassify cases involving underrepresented or marginalized groups, leading to missed interventions and biased police responses (Bland, 2020).

A potential way to mitigate these risks is selective classification, which allows predictive systems to abstain when confidence in a prediction falls below a specified threshold (Geifman and El-Yaniv, 2019). This auto-rejection mechanism typically improves model performance by avoiding low-confidence decisions, though at the cost of reduced coverage. In operational settings, human-in-the-loop workflows can be used to defer these abstentions to expert review (Butcher et al., 2024). While a combination of text mining and machine learning classifiers has previously been used to extract and process information from police-recorded crime data (Adily et al., 2021; Birks et al., 2020; Karystianis et al., 2019; Victor et al., 2021), to the best of our knowledge, a selective classification strategy has not yet been explored for extracting information from free-text DVA records.

We sought to address this gap by developing two machine learning algorithms to extract victim-offender relationship information from a dataset of DVA incidents obtained from a single UK police force. We first experimented with their ability to classify ex-partner, current partner, and familial cases of DVA without abstention (i.e., a conventional supervised machine learning framework). We then compared the performance to selective classification models that could reject a prediction when uncertain. This study builds upon existing research by demonstrating the feasibility of selective classification for extracting relationship data from free-text DVA police records, highlighting both its potential to improve data quality and its implications for risk assessment and operational decision-making.

## Method

### Dataset

Guidelines for how police forces record crime in England and Wales are governed by Home Office Counting Rules (HOCR). An incident is considered a crime if a report of criminal activity has been made to the police and attending officers uphold the view a crime has occurred based on the presence of a victim and lack of evidence to the contrary. Crimes are recorded per victim (i.e., multiple incidents are recorded if a crime has more than one victim), and in the event multiple crimes occur within a sequence, the most serious offense is recorded. The classification of a crime is made by the attending officer (Home Office, 2025).

As noted by Phoenix (2023), DVA is not defined as a single statutory offense under UK law. Consequently, it does not have its own classification code designated by the Home Office. Instead, HOCR requires police forces to manually flag incidents suspected of DVA on the basis the victim and perpetrator are personally connected (Home Office, 2025). This manual flagging process, in effect nationwide since 2015, has been linked to inconsistencies in how DVA is recorded across regions, contributing to known issues in data quality, under-reporting, and cross-force comparability (HMICFRS, 2019).

With this in mind, we acquired DVA incident data from a single UK police force with a high-level of reported crime recording accuracy (Whitehead, 2024). The data, comprising DVA incidents recorded between 2020 and 2024, was provided by the host force as a comma-separated values (CSV) file. Sharing of incident records were subject to a data sharing agreement between the University of Lancashire, and the host force.

The data file consisted of 19,013 rows and 31 columns. Each row reflected a separate DVA incident. Columns comprised structured data fields including crime reference information and demographic details about the victim and the suspected perpetrator, including their age, ethnicity, gender, and relationship type. Columns also flagged the suspected presence of alcohol, drugs, firearms, and knives, and whether an arrest had been made. The final column, titled *Crime Notes*, included a free-text description of the incident. All data was fully de-identified by the host force as a condition of access.

### Crime notes

**Figure 1:**
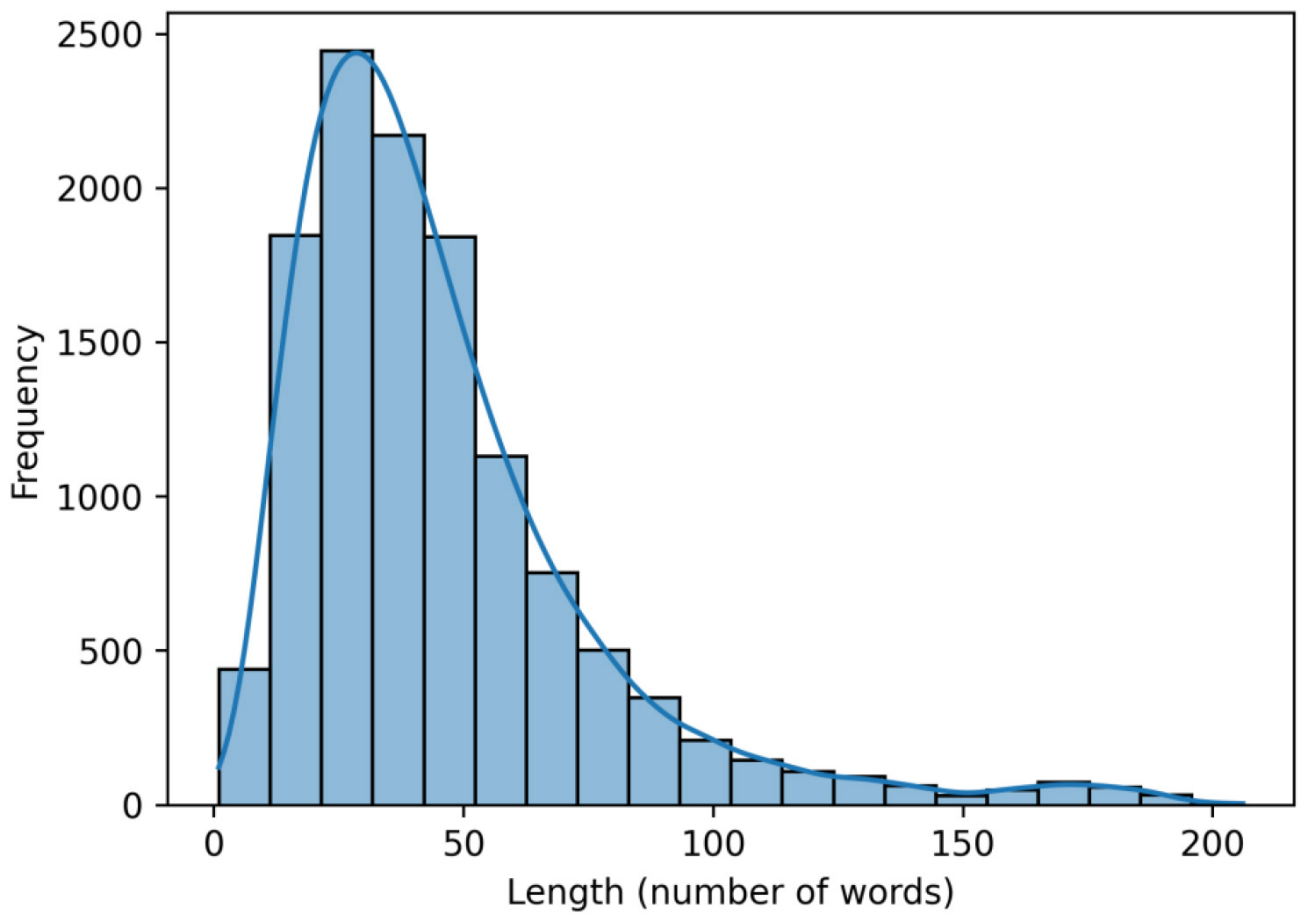
Distribution of the length of crime notes by the number of words.

The crime notes column formed the primary text corpus for model training and evaluation. These notes are written by attending officers at the time of the incident and consist of brief telegraphic descriptions that include a large amount of police terminology and abbreviations. The average length of a note was 46 words (median = 38; SD = 31), and the longest was 206 words. Variations in length (see Figure 1) result in crime notes that differ considerably in their semantic richness and lexical diversity, with many containing short snippets of information while others provide more detailed accounts of the incident.

### Data filtering

We first filtered the dataset to remove records likely to contribute noise, ensuring that the machine learning model was trained only on incidents with valid labels and meaningful free-text descriptions. Given our focus on extracting the relationship type from free-text, we removed all columns aside from the relationship type and crime notes. We then removed all rows where either variable was blank.

We excluded any rows where the crime notes contained duplicated or boilerplate phrases, as these can contribute to *data leakage*—a concept within machine learning where information about the target variable is inadvertently made available to the model during training (Kaufman et al., 2012). Data leakage essentially allows the model to cheat, artificially inflating performance and leading to an overestimation of how well it will generalize to new, unseen inputs, ultimately reducing its effectiveness in real-world settings (Zimmermann et al., 2023).

Lastly, we removed rows where the relationship type did not meet the Home Office (2025) definition of DVA and may have been flagged in error. For instance, where the suspect was recorded as an acquaintance of the victim, or where the victim declines to identify the suspect to police. After these filtering steps, our dataset comprised 14,119 confirmed DVA incidents, representing 74% of the initial dataset.

### Labeling schema for victim/offender relationship type

Our dataset included 56 unique combinations of tags to describe the relationship between the victim and the offender. Officers could input multiple relationship tags to indicate an incident with more than one suspect, although 98% of our raw data only referenced a single offender. Reflecting national DVA trends (Long and Harvey, 2020), ex-partner was the most common relationship in the raw data (40%), followed by current boyfriend/girlfriend (21%) or variations thereof (e.g., spouse) (9%). Other tags were included that related to the wider family unit, such as parent-of-offender (10%), sibling-of-offender (5%), and child-of-offender (3%).

Supervised machine learning performs best when boundaries between classes are semantically distinct. Linguistic overlap or ambiguity between classes (e.g., boyfriend/girlfriend and spouse) can increase label noise and contribute to misclassifications (Frenay and Verleysen, 2014). A classification task constructed around non-fuzzy boundaries is therefore desirable whenever possible.

Furthermore, we observed substantial class imbalance in the raw data. The most common tag, “ex-partner,” accounted for 40% of all DVA incidents, whereas “child-of-offender” made up only 3% of incidents. Whilst a degree of imbalance is inevitable in non-laboratory settings, reflecting real-world variability, large class disparities can reduce classification performance by training the model to overpredict the dominant class while failing to learn enough to accurately predict minority classes (He and Garcia, 2009).

We sought to resolve both problems by simplifying the labeling schema to three classes. As the dominant tag, “ex-partner” was not altered. We aggregated tags for “boyfriend/girlfriend” and “spouse” into a second class named “current partner,” analogous to intimate partner violence (IPV). Lastly, we grouped tags related to other family members, such as “child-of-offender” and “parent-of-offender” into a third class named “family.” Any remaining rows (482), which included non-family relations (e.g., neighbors, employees), were inspected and removed from our dataset. We illustrate the revised data distribution for this simplified schema in Table 1.

**Table 1:** Distribution of relationship type by class label.

**Class label**	**Freq**.	**Perc. (%)**
Ex-partner	5,828	46.7
Current partner	4,156	33.3
Family	2,502	20.0

Most (46.7%) DVA incidents in our final dataset were committed by an ex-partner, compared to 33.3% by a current partner, and 20% by a member of the victim's family.

### Supervised machine learning

Supervised machine learning is a method for learning a mathematical function *f*(*X*) that can predict an output variable *Y*–in this case *the relationship type*—based on an input *X*, the content of the police crime notes (Leitgöb et al., 2023). The model works by iteratively evaluating known pairs of *X* and *Y* to learn a set of parameters that minimize prediction error, thereby enabling accurate predictions on unseen data (Molina and Garip, 2019). The goal in this process is to strike a balance between fitting the training data too closely, including its noise (overfitting), and fitting it too loosely, which increases error on the training data itself (underfitting). A standard way to check this balance is to divide the dataset into separate regions for training and testing. In our case, we allocated 70% of the data to training the model and 30% to testing its performance. To ensure that each set contained a proportional representation of each relationship class, we used a stratified splitting approach via scikit-learn's train_test_split function, with the stratify parameter set to the class label. We also set a fixed random seed to ensure a reproducible split.

#### Preprocessing the content of the police crime notes

Machine learning models do not interpret raw text in the same way humans do. Instead, they need to be represented in a numerical format (Grimmer et al., 2022). An example of representing text numerically is to define each word in a document by its frequency of occurrence. Words with the highest relative frequency represent a description of the document, following Firth's (1957, p. 11) distributional hypothesis that “*you shall know a word by the company it keeps*.” Despite their simplicity, these methods have been shown to yield results that match or exceed more sophisticated approaches in information retrieval and document classification tasks (Salton and Buckley, 1988). More recent innovations, such as word or document embeddings, map text into dense numerical vectors that capture semantic relationships between terms (Mikolov et al., 2013). Regardless of the method, modeling text numerically typically produces high-dimensional and often sparse vector spaces, making preprocessing steps critical for reducing dimensionality and model complexity Uysal and Gunal, (2014).

We followed a series of steps outlined by Hickman et al. (2022) to preprocess the crime notes before training our machine learning models. First, we converted all text to lowercase, ensuring that the word tokens “crime” and “Crime” are considered equivalent, thereby increasing the model's statistical power. We also removed non-alphabetic characters such as punctuation and numbers, and any additional whitespace between words.

We then automatically corrected misspellings using Levenshtein Distance, which measures the minimum number of single-character edits (insertions, deletions, or substitutions) needed to transform one word into another (Levenshtein, 1966). If a word did not appear in a standard English dictionary, it was replaced with the closest match if the Levenshtein Distance was less than two edits. If no such match existed, we retained the original misspelling.

#### Machine learning algorithms

Algorithms are the specific rules that govern how a model processes information and makes decisions (Cormen et al., 2022). The choice of algorithm depends on several factors, including the size and type of data, as well as the available computational resources. To evaluate the effectiveness of a chosen model, it is common to compare its performance against a simple, well-understood baseline estimator, which serves as a reference point (Géron, 2022). In this work, we compare the performance of two machine learning algorithms (Random Forest and DistilBERT^1^) to a simple rule-based estimator, which acted as our baseline model.

##### Random forest

A random forest is an ensemble learning method that builds multiple decision trees, each trained on a bootstrap sample of the training data. At each branching point within a tree, a random subset of features (in this case, words from the crime notes) is considered, which helps make the trees more diverse. A prediction about an input is obtained by aggregating the output over all trees through majority voting (Breiman, 2001).

To train the random forest, we represented words within crime notes as numerical features using the Term-Frequency/Inverse-Document-Frequency (TF-IDF) method. TF-IDF is a feature engineering technique that assigns higher weights to words that appear frequently in a document relative to the remainder of a corpus (Salton and Buckley, 1988), serving as a numerical representation of the *importance* of a particular term. We built 100 decision trees and used the default hyperparameters implemented by scikit-learn, including tree depth (unlimited, no pruning), the minimum number of samples to split a node (2), and the minimum number of samples required at a leaf node (1) (Pedregosa et al., 2011).

##### DistilBERT

DistilBERT is a transformer-based deep learning model for natural language processing, derived from BERT, but with fewer parameters for faster inference (Devlin et al., 2019; Sanh et al., 2020). Unlike random forests, which consider each word as an individual feature, DistilBERT analyses the whole sequence of input text using token embeddings, enabling it to capture context and meaning more easily than a bag-of-words model. During training, these embeddings are processed through multiple transformer layers, with the final layer producing a logit score for each possible class label. Logits are converted to probabilities via the Softmax function, and the label with the highest probability is selected as the model's prediction.

We fine-tuned the pre-trained distilbert-base-uncased model from the Hugging Face Transformers library (Wolf et al., 2020) using the data in our training set. Text was tokenized into subword units, converted to token IDs, and padded or truncated to a maximum sequence length of 256 tokens. The model was fine-tuned for three epochs using the AdamW optimizer (Loshchilov and Hutter, 2019) with a learning rate of 5 × 10^−5^ and a batch size of 8. The model's output layer was adapted to the three class labels in our dataset.

##### Rules-based (baseline)

Our baseline model was a rules-based classifier that generates predictions using a set of static, predefined rules based on the presence of specific keywords in the crime notes (see Algorithm 1 for an illustration). For example, crime notes containing “ex-boyfriend” or “divorce” are assigned to the ex-partner class label. These rules were defined by the authors and rely on words that are strongly associated with each relationship type. If the crime notes do not contain a class-specific keyword, they are automatically classed as “ex-partner,” as it was the most frequent class.

Algorithm 1 Pseudocode describing how crime notes are used by the baseline estimator to assign a class label based on the presence of keywords.

 for each **note** in **crime_notes**:
  if **note** contains any **ex_partner_keywords**:
   **label** = “**ex_partner**”
  else if **note** contains any
 **current_partner_keywords**:
   **label** = “**current_partner**”
  else if **note** contains any
 **family_keywords**:
   **label** = “**victims_family**”
  else:
   **label** = “**ex_partner**”

While simple and transparent, this approach is limited to detecting patterns explicitly defined a priori, and may overlook unexpected, subtle or less direct language. Our choice of a rules-based model as our baseline was influenced by prior studies that have successfully used syntax-based rules to extract information from crime reports (Victor et al., 2021; Karystianis et al., 2019).

### Selective classification

A limitation of conventional supervised machine learning is that a prediction is generated for every input, even when the input contains no relevant information for the task. In our context, this can lead to invalid classifications when the crime notes contain no reference to the relationship type. In practice, especially given the inherent high-risk of crime recording, we would want to avoid making predictions where it is not suitable to do so.

To resolve this, we include an abstention function after prediction. The model produces a confidence score for each class, and if the highest score falls below a predefined threshold τ (e.g., 70%), the prediction is withheld (see Algorithm 2). Increasing τ makes the model more conservative, generating predictions only when confidence is high. This approach should improve precision by reducing false positives, though at the cost of lower coverage (the proportion of the test set for which a prediction is made; Chow, 1970).

Algorithm 2 Pseudocode describing how the model abstains when confidence is below a given threshold (τ).

 for each **note** in **crime_notes**:
  **predicted_label**, **confidence** = model.predict(**note**)
  if **confidence** >= τ:
   output = **predicted_label**
  else:
   output = “**no prediction**”

### Evaluation strategy

We evaluate the performance of each model by using a confusion matrix to compare the predictions to the actual values (i.e., the ground truth) in the test set. We use precision, recall, and F1-score as our evaluation metrics. We avoid using accuracy due to the between-class imbalance in our data that would disproportionately influence results toward the performance of the majority class.

#### Precision

Precision is the proportion of predicted positive classifications that were correct. For instance, predicting “ex-partner” when the correct value was in fact “ex-partner” is a *true positive* (TP), whereas an incorrect prediction would be a *false positive* (FP). A high precision score (max score = 1) means that the model made fewer false positive errors:


$$\begin{array}{l} precision=\frac{TP}{TP+FP} \end{array}$$

#### Recall

Recall, also known as the true positive rate or *sensitivity*, is the proportion of actual positives in the test set that were correctly predicted by the model. A false negative (FN) is where the model should have predicted a given class (e.g., “ex-partner”) but failed to do so:


$$\begin{array}{l} recall=\frac{TP}{TP+FN} \end{array}$$

#### F1-score

F1-score is the harmonic mean of precision and recall. Compared to accuracy, it is a more suitable measure of overall performance when working with imbalanced classes:


$$\begin{array}{l} F1=2\times \frac{precision\times recall}{precision+recall} \end{array}$$

**Figure 2:**
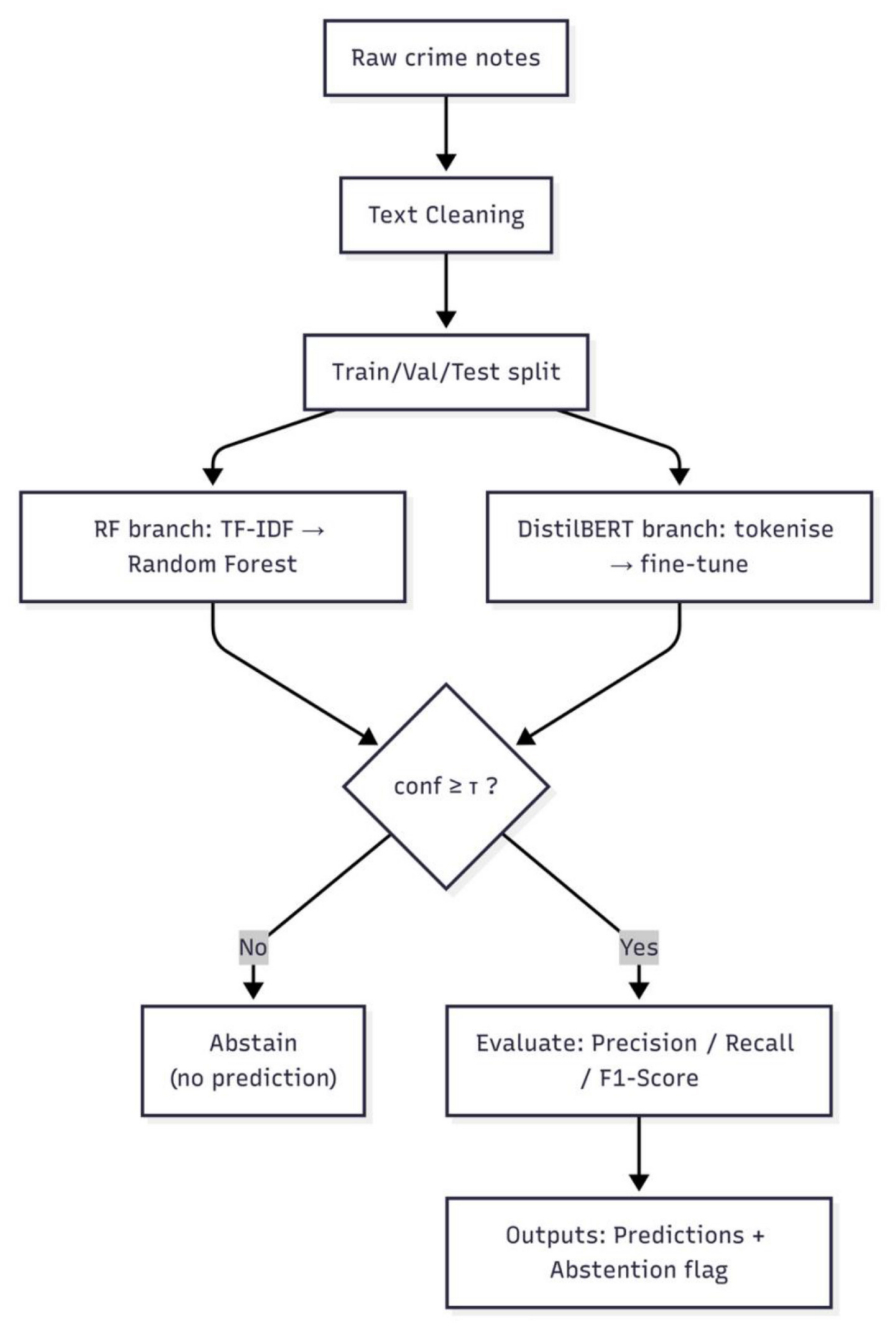
Overview of the pipeline, illustrating data cleaning, splitting, training, confidence estimation, and prediction steps.

A schematic overview of the full pipeline, from data preprocessing through model training, evaluation, and selective classification, is shown in Figure 2.

## Results

### Overall model performance—ML approaches outperform the baseline

When applied to the full test set without abstention, both machine learning models outperform the baseline. DistilBERT achieved the highest overall performance (Precision = 83%, Recall = 82%, F1 = 82%), followed by Random Forest (Precision = 79%, Recall = 78%, F1 = 78%), compared to the baseline's lower scores (Precision = 70%, Recall = 64%, F1 = 66%). See Table 2 for a performance breakdown.

**Table 2:** Performance (Precision, Recall, F1) of each machine learning model (Random Forest and DistilBERT) and the baseline (Rule-Based) when predictions are made on the full test set (without abstaining).

**Model**	**Precision (%)**	**Recall (%)**	**F1 (%)**
Rule-based	70	64	66
Random Forest	79	78	78
DistilBERT	83	82	82

These results represent improvements over the baseline of +9% precision and +14% recall for random forest, and +13% and +18% recall for DistilBERT. In practical terms, a 13% gain in precision for DistilBERT would prevent around 130 false classifications for every 1,000 incidents processed. In a police force handling 5,000 DVA incidents annually, this could result in 650 fewer relationship-type errors each year.

The better performance of DistilBERT likely reflects its ability to interpret context within the crime notes, for example, by recognizing whether the word “partner” is modified by time cues like “former” or “still living with.” In contrast, Random Forest with TF-IDF treats words independently, making it more vulnerable to misclassification in ambiguous cases.

Notably, even the lower-performing ML model (RF) still showed large gains over the baseline, suggesting that either approach could enhance the accuracy of DVA relationship classification in operational settings. Given the safeguarding implications of incorrect classifications, both models offer potential to reduce investigative risk and improve resource targeting.

### Class-level trends—“Current-partner” is the hardest relationship to predict

Across classes, both ML models found ex-partner easiest to identify, achieving F1 scores of 82% (Random Forest) and 85% (DistilBERT; see Table 3). This is unsurprising given that 47% of training cases were labeled ex-partner, and the crime notes often contained unambiguous terms such as “ex-husband” or “former boyfriend.” DistilBERT achieved balanced precision (85%) and recall (85%), while Random Forest showed slightly more false positives, with lower precision (78%) despite higher recall (86%).

**Table 3:** Class-level performance (Precision, Recall, F1-score) of each machine learning model (Random Forest and DistilBERT).

**Class**	**RF Precision (%)**	**DB Precision (%)**	**RF Recall (%)**	**DB Recall (%)**	**RF F1 (%)**	**DB F1 (%)**
Ex-partner	78	85	86	85	82	85
Current partner	71	73	78	82	75	77
Family	91	93	64	77	75	84

**Figure 3:**
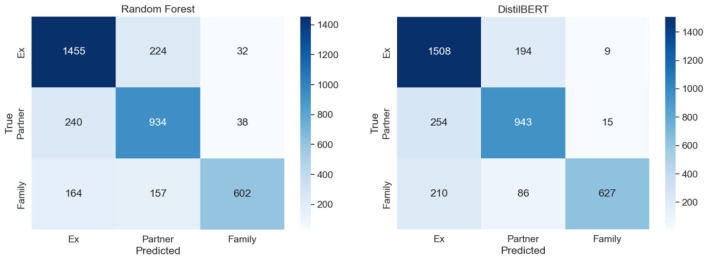
Confusion matrix showing the differences in class-wise classification and misclassification between Random Forest **(left)** and DistilBERT **(right)**.

In contrast, current partner was the most challenging class, with F1 scores of 75% (Random Forest) and 77% (DistilBERT). Precision was notably lower here (RF = 71%, DB = 73%), reflecting frequent misclassification of these cases. A visual inspection of the misclassified crime notes, alongside the confusion matrix (see Figure 3), shows this often occurred when the input text referenced phrases like “partner” or “boyfriend” without specifying whether the relationship had ended (e.g., “her partner had previously assaulted her”). Such wording requires contextual interpretation that may exceed the capacity of a bag-of-words model like Random Forest and can still challenge DistilBERT when temporal cues are ambiguous.

For the family class, both models achieved very high precision (RF = 91%, DB = 93%), but recall was substantially lower for Random Forest (64%) compared to DistilBERT (77%), creating an F1 gap of 9% points (75% vs. 84%). This finding suggests DistilBERT was better able to detect familial relationships even when family terms appeared in more complex contexts. For example, “her brother, who lives nearby, was involved in the dispute.”

### Selective classification improves precision, especially for the “current-partner” class

Allowing the models to abstain when prediction confidence was low substantially increased precision, with the largest gains seen for the current partner class. At a confidence threshold (τ) set to achieve 60% coverage (comparable to the baseline's natural abstention rate of 40%), precision for the current partner class rose by an average of +15% points across the two models (see Table 4). In practical terms, for a dataset containing 500 current-partner cases, this gain would prevent approximately 75 false classifications.

**Table 4:** Precision performance of each machine learning model (Random Forest and DistilBERT) when the model could abstain if uncertain (60% coverage).

**Class**	**RF No Abstain (%)**	**RF Abstain (%)**	**DB No Abstain (%)**	**DB Abstain (%)**
Ex-partner	78	89	85	92
Current partner	71	83	73	91
Family	91	97	93	96

Overall, the selective classification strategy improved average precision by +9.7% for Random Forest and +9.3% for DistilBERT. Gains for the easier-to-detect classes (ex-partner and family) were smaller but still positive, reflecting the finding that these categories were already well-classified without abstention. The largest benefits came from filtering out ambiguous records, for example, notes stating “her partner, who she used to live with,” or “the suspect is a family friend,” where relationship type cannot be inferred with high certainty from text alone.

However, these precision gains came at the cost of reduced coverage, with 40% of cases left unclassified at the chosen τ. In operational DVA settings, this trade-off may still be advantageous, as unclassified cases can be flagged for manual review by experts, thus avoiding the risk of incorrect automatic classification that could misinform safeguarding measures. The biggest improvement was observed in the current partner class (an average improvement of 15% across the two models), indicating that abstention provides an efficient way to improve model performance by rejecting hard-to-classify instances.

### Precision-coverage trade-offs differ between ML models

**Figure 4:**
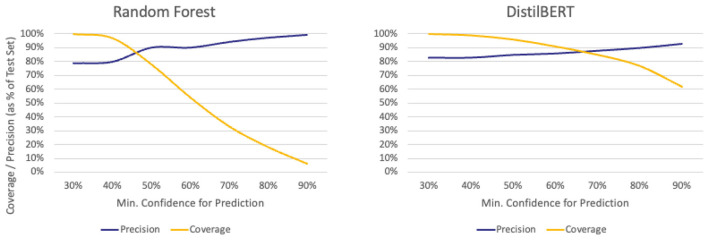
Comparison of precision and coverage statistics for each machine learning model at different values of τ (*x*-axis). Lower values on the *x*-axis indicate a more lenient model, whereas higher values indicate a stricter model. Coverage (orange line) decreases as τ increases. The blue line illustrates changes in precision at different values of τ.

Raising τ improved precision for both models but reduced the proportion of cases classified. DistilBERT maintained far higher coverage under strict settings: at τ=90%, it still classified around 60% of cases, compared to less than 10% for Random Forest (see Figure 4). For a force processing 5,000 incidents annually, this would mean DistilBERT could automatically classify about 3,000 cases at this setting, while Random Forest would classify fewer than 500, leaving the rest for manual review.

This divergence in performance reflects differences in how the models generate confidence scores. DistilBERT's contextual embeddings often produce higher certainty when patterns are recognized, even with complex phrasing, whereas Random Forest's word-based features can yield lower confidence in cases where the wording is unfamiliar or partially missing.

From an operational standpoint, this means DistilBERT is better suited when large volumes of data must be processed and the cost of false positives at this scale is high, as it can remain conservative without discarding most cases. Random Forest's steep drop in coverage after τ=40% makes it more appropriate when interpretability and transparency are priorities. Examples of this might include instances where analysts are required to justify each automated decision or low-volume cases in which manual verification becomes a practical alternative.

## Discussion

Our findings demonstrate that machine learning can substantially improve the accuracy of crime recording of victim–offender relationship types in DVA cases. Both models we tested, a Random Forest and a fine-tuned DistilBERT model, outperformed a rule-based baseline across all performance measures, with DistilBERT achieving the highest overall precision (83%), recall (82%), and F1-score (82%). These gains result in more reliable relationship data in at least four out of five cases, reducing the manual effort required by analysts and improving the completeness of police crime records.

Performance metrics varied by relationship category. Ex-partner cases were classified most accurately, while current-partner cases proved more challenging. We suspect this is mainly due to linguistic overlap in the crime notes. Introducing a selective classification approach, which allows the model to abstain when confidence is low, boosted precision for “current partner” cases by approximately 15%, albeit at the cost of reduced coverage. Our findings highlight the potential for computational tools to address long-standing data quality issues in policing (see Whitehead, 2024) and to do so in a way that balances accuracy with operational safety.

These findings are broadly consistent with, and extend, a growing body of research applying machine learning and text mining to extract information from DVA narratives (Karystianis et al., 2019; Adily et al., 2021; Neubauer et al., 2023). In particular, one study used supervised machine learning to identify instances of DVA in caseworker reports and found that a statistical model (k-nearest neighbor) outperformed a rule-based approach (Victor et al., 2021). Our work extends this line of research in two ways. First, we apply more recent algorithms, including a transformer-based model, to the related but distinct task of classifying victim–offender relationships, and second, we do so on substantially shorter narratives. Whereas the aforementioned study analyzed documents with a minimum length of 50 words, the average crime note in our dataset was only 46 words, with very few exceeding 100 words. Short-text classification is known to be more challenging for machine learning models due to the presence of sparse features and limited contextual cues (Song et al., 2014; Wang et al., 2017), making the observed performance gains here particularly noteworthy.

Our finding that the “current partner” class was the most difficult class to identify, with lower precision than both “ex-partner” and “family” classes, reflects the ambiguity in how police describe these relationships in crime notes. This result aligns with prior work showing that even slight variations in how DVA incidents are described can cause difficulties for both human annotators and machine classifiers (Victor et al., 2021). In our data, we observed substantial lexical overlap between the ex-partner and current partner classes, a challenge also noted by Beigman-Klebanov and Beigman (2010), who found that subtle lexical substitutions can reduce classification accuracy for both experts and automated systems. Class imbalance may have compounded this issue, as outlined by He and Garcia (2009), by limiting the number of distinctive current partner examples available during training. One possible way to mitigate this would have been the use of weighted random sampling, which could have helped the model place greater emphasis on underrepresented examples. The relatively higher performance for the “ex-partner” class likely reflects both its greater prevalence and the presence of clear lexical markers, such as “ex-,” “former,” or “divorce.” While synthetic oversampling techniques like SMOTE could potentially address class imbalances, these methods risk amplifying noise and overfitting in text-based models (Chawla et al., 2002). For the “family” class, DistilBERT's higher recall suggests that contextual embeddings may be better equipped to detect the broader set of familial relationships that are mentioned indirectly or embedded within more complex narrative structures.

We also find evidence to support the value of a selective classification approach as a precision-enhancing strategy in high-stakes applications. By allowing the models to withhold predictions when confidence was low, we achieved precision gains of around 15% when classifying “current partner,” the most challenging class, and smaller but still positive gains in the other categories. These findings are consistent with previous studies that argue that abstention improves decision reliability in domains where the cost of misclassification is high (Chow, 1970; Geifman and El-Yaniv, 2019). In our context, we observed an increase in precision, a reduction in false positives, by automatically filtering out records with ambiguous cues (e.g., “her partner, who she used to live with”), which are difficult to classify correctly, a finding that aligns with Butcher et al. (2024). While this approach reduces coverage, the trade-off is operationally acceptable, as cases without a confident prediction can be flagged for human review, avoiding potentially harmful misclassifications in safeguarding and investigative contexts.

The trade-off between precision and coverage highlights important differences between our two machine learning models. DistilBERT retained substantially higher coverage at strict confidence thresholds, continuing to classify around 60% of test cases even when the minimum acceptable confidence was set to 90% certainty. Comparatively, the Random Forest, at the same confidence level, classified less than 10% of the available data. This difference likely reflects DistilBERT's ability to utilize contextual information to make confident predictions, even when explicit relationship markers are not present. In practice, this makes DistilBERT the better option when large datasets must be processed with minimal false positives. Conversely, Random Forest, with faster training times and greater transparency, may be preferable on smaller datasets, or when model interpretability is paramount (Arhiliuc and Guns, 2023).

### Limitations

As noted by Karystianis et al. (2019), our models are reliant on the host force's internal quality checks to ensure the accuracy of the provided data. If the underlying records are inaccurate, prediction quality will inevitably suffer (Turner et al., 2019). In addition, due to privacy and confidentiality constraints when working with police data, such issues may go undetected. While our use of a held-out test set helps flag poorly performing models, assuming no data leakage (Kaufman et al., 2012), this safeguard is limited by the representativeness of the test data. Sensitivity checks, where we experiment with different data-splitting strategies (e.g., 60/40, 70/30, 80/20) are one solution to this challenge, although were not undertaken here due to time constraints. A more robust alternative is *K*-fold cross-validation (Kohavi, 1995), which repeatedly trains and tests the model on different subsets of the data, reducing the risk of sampling bias. This too was deemed impractical due to the high computational cost of applying such methods to deep learning models. With our current setup, fine-tuning DistilBERT for three epochs^2^ on a single train–test split required approximately 35 min using a MacBook Pro with an M1 chip. Applying *K*-fold cross-validation would have increased total training time to many hours. Future work could enhance confidence in our initial findings by experimenting with different splitting and training strategies using dedicated GPU resources or high-performance computing infrastructure.

The single origin of our data limits the generalizability of our results beyond the individual force. To address this, we plan to triangulate our findings with data from other UK forces in future work. Given the challenges of acquiring police data in sufficient volume to adequately train machine learning models, complementary approaches such as text augmentation (Wei and Zou, 2019) and adversarial test cases (Morris et al., 2020) provide an alternative way to evaluate robustness and generalizability beyond the initial held-out set. A further practical consideration is that 26% of our dataset could not be used due to missing, duplicated, or inconsistent entries. While we removed these datapoints to ensure a robust and leakage-free model, they highlight the importance of improving the overall quality and completeness of administrative records within policing.

While our use of selective classification provides a practical means of enhancing precision by filtering uncertain predictions, it has notable limitations. The threshold for determining whether a prediction is sufficiently confident is model-dependent and may therefore need to be redefined when applied to different architectures or datasets. Moreover, selective classification does not resolve the underlying difficulty of ambiguous cases but improves performance by setting aside instances that are less reliably classified. In our context, however, this strategy was justified, as not all crime notes necessarily contained an explicit reference to the relationship type. Because of this, some cases should have been impossible to classify with confidence, irrespective of model performance. We view selective classification as a pragmatic strength in contexts where reducing false positives is especially important. From a practical standpoint, it was preferable to leave an input unclassified than to risk a false classification, particularly given the resource or procedural implications that may follow from mislabeling a victim incorrectly. Nevertheless, in applications where full coverage is required, complementary approaches such as targeted human annotation or expert validation of hard-to-classify cases could help address these challenges without relying solely on an abstaining model.

### Ethical considerations: fairness and bias

Because our models are trained on police crime notes, their performance is shaped by the way officers record incidents. Any reporting inconsistencies along societal-level boundaries (e.g., differences in race, ethnicity, sexuality, or migrant status) risk being carried through into the model during training, potentially leading to models that are better calibrated for majority cases than for minoritised groups (Barocas et al., 2023). Disparities in representativeness, particularly within policing, can be difficult to identify and overcome (Lum and Isaac, 2016). In a preliminary inspection of our dataset, we observed that a high majority of victim ethnicity data was recorded as white. This imbalance may reflect the underlying distribution of reported cases, but it could also be compounded by incomplete or missing entries for non-white victims. With the current data, we cannot determine the pattern or causes of missingness and thus cannot reliably assess whether the models perform differently across demographic groups. Accordingly, fairness-related disparities cannot be ruled out.

### Impact on policing

This study demonstrates that machine learning can be used to automatically fill in missing victim–offender relationship data in DVA cases with a high degree of accuracy, potentially improving the completeness of police crime records while reducing analyst workload. Our findings have direct operational benefits, generating more complete and accurate relationship data that can strengthen safeguarding assessments, inform investigative decisions, and improve the evidence base for policy (Whitehead, 2024).

However, these benefits depend on having sufficiently high-quality, representative data for the target variable. In our study, relationship type was a suitable candidate because it was both operationally important and sufficiently well-represented in police records to train effective models. When we tested the same approach on variables with very low variation, the model's performance dropped to nearly zero. Under these conditions, conventional supervised machine learning offers little operational value without additional data collection or careful rebalancing of the dataset.

Compared to conventional classification tasks, where an output is generated for each input, our selective classifier, with its reduction in coverage, reflects a deliberate architectural design choice. Rows where the victim–offender relationship type was absent from the crime notes were excluded, since the model cannot, *and should not*, predict information that is not present. In an operational setting, these no-prediction cases could be routed to expert review. A human-in-the-loop approach, where uncertain or incomplete inputs are presented to users via an interface, can significantly increase coverage while maintaining efficiency and precision (Butcher et al., 2024). Deference to a human verifier also provides an explicit opportunity to confirm when the free text does not contain the requisite information, thereby ensuring that the system complements, rather than replaces, professional judgment.

For policing more generally, the key message is that machine learning offers a viable route to targeted improvements in data quality, but it is not a one-size-fits-all solution. Before deploying such tools in live systems, forces should assess the distribution and quality of the data for the intended variable and consider extensive piloting of the approach in a controlled environment. Without such safeguards, there is a risk that biased or incomplete data could lead to misleading outputs and reinforce existing disparities (Lum and Isaac, 2016). Further research into approaches that can handle extreme imbalance will be essential if this technology is to be applied more broadly across police datasets.

### Conclusions

In this study, we evaluated the capability of supervised machine learning models to automatically extract victim–offender relationship information from free-text crime notes in DVA cases. Both models demonstrated that such tools could serve as cost-effective and efficient alternatives to manual coding, accurately classifying relationship type in around four out of five cases. This finding represents a meaningful step toward addressing long-standing concerns about the completeness and reliability of police-recorded crime data (Whitehead, 2024). Given that police-recorded crime lost its status as an accredited official statistic in 2014, the application of data science methods to reliably impute missing values offers a promising route to restoring confidence in these records.

The incorporation of a selective classification function improved precision for the most challenging cases by abstaining from low-confidence predictions, though at the cost of reduced coverage. Future work should explore more advanced uncertainty quantification methods (Zhang et al., 2023) and human-in-the-loop designs (Butcher et al., 2024) to maintain high precision while improving coverage. Such developments could support operational decision-making in ways that are both data-driven and safeguard-conscious, enabling police to make better use of the information they already collect.

## Data Availability

The dataset presented in this article is not readily available due to conditions outlined in the data sharing agreement with the host force. Requests to access the datasets should be directed to darren.cook@city.ac.uk.

## Footnotes

1 Other architectures were considered in this work. However, we opted for DistilBERT as our deep learning model as it is substantially smaller than BERT while retaining strong performance.

2 The authors wish to note that the decision to use three epochs reflected a computational trade-off instead of an exhaustive search for optimal performance.

## References

[B1] Adily A. Karystianis G. Butler T. (2021). Text Mining Police Narratives to Identify Types of Abuse and Victim Injuries in Family and Domestic Violence Events (No. 630). Canberra, ACT: Australian Institute of Criminology. doi: 10.52922/ti04923

[B2] Arhiliuc C. Guns R. (2023). “Content-based classification of research articles: comparing keyword extraction, BERT, and random forest classifiers,” in ISSI 2023: the 19th International Conference of the International Society for Scientometrics and Informetrics (ISSI2023), 2-5 July, 2023 (Bloomington, IN), 4363.

[B3] Barocas S. Hardt M. Narayanan A. (2023). Fairness and Machine Learning: Limitations and Opportunities. Cambridge, MS: MIT Press.

[B4] Barrow-Grint K. Sebire J. Turton J. Weir R. (2022). Policing Domestic Abuse: Risk, Policy, and Practice. Oxfordshire: Routledge. doi: 10.4324/9781003137412

[B5] Beigman-Klebanov B. Beigman E. (2010). “Some empirical evidence for annotation noise in a benchmarked dataset,” in Human Language Technologies: The 2010 Annual Conference of the North American Chapter of the Association for Computational Linguistics, eds. R. Kaplan, J. Burstein, M. Harper, and G. Penn (Association for Computational Linguistics), 438–446. Available online at: https://aclanthology.org/N10-1067/ (Accessed August 13, 2025).

[B6] Birks D. Coleman A. Jackson D. (2020). Unsupervised identification of crime problems from police free-text data. Crime Sci. 9:18. doi: 10.1186/s40163-020-00127-4

[B7] Bland M. (2020). “Algorithms can predict domestic abuse, but should we let them?” in Policing in the Era of AI and Smart Societies, eds. H. Jahankhani, B. Akhgar, P. Cochrane, M. Dastbaz and Sierra C. (Cham: Springer International Publishing), 139–155. doi: 10.1007/978-3-030-50613-1_6

[B8] Blom N. Obolenskaya P. Phoenix J. Pullerits M. (2024). Physical and emotional impacts of intimate partner violence and abuse: distinctions by relationship status and offence type. J. Fam. Viol. doi: 10.1007/s10896-024-00786-w

[B9] Breiman L. (2001). Random forests. Mach. Learn. 45, 5–32. doi: 10.1023/A:1010933404324

[B10] Butcher B. Zilka M. Hron J. Cook D. Weller A. (2024). Optimising human-machine collaboration for efficient high-precision information extraction from text documents. ACM J. Respons. Comput. 1, 1–27. doi: 10.1145/3652591

[B11] Chawla N. V. Bowyer K. W. Hall L. O. Kegelmeyer W. P. (2002). SMOTE: synthetic minority over-sampling technique. J. Artificial Intell. Res. 16, 321–357. doi: 10.1613/jair.953

[B12] Chow C. (1970). On optimum recognition error and reject tradeoff. IEEE Trans. Information Theory 16, 41–46. doi: 10.1109/TIT.1970.1054406

[B13] Cormen T. H. Leiserson C. E. Rivest R. L. Stein C. (2022). Introduction to Algorithms. Cambridge, MS: MIT Press.

[B14] Devlin J. Chang M.-W. Lee K. Toutanova K. (2019). “BERT: pre-training of deep bidirectional transformers for language understanding,” in Proceedings of the 2019 Conference of the North American Chapter of the Association for Computational Linguistics: Human Language Technologies, Vol. 1: Long and Short Papers, eds. J. Burstein, C. Doran, and T. Solorio (Minneapolis, MN: Association for Computational Linguistics), 4171–4186.

[B15] Domestic Abuse Act C. 17 §, Part, 1. (2021). Available online at: https://www.legislation.gov.uk/ukpga/2021/17/part/1 (Accessed August 15, 2021).

[B16] Firth J. (1957). A synopsis of linguistic theory, 1930-1955. Stud. Linguistic Anal. 10–32.

[B17] Frenay B. Verleysen M. (2014). Classification in the presence of label noise: a survey. IEEE Trans. Neural Netw. Learning Syst. 25, 845–869. doi: 10.1109/TNNLS.2013.2292894 24808033

[B18] Geifman Y. El-Yaniv R. (2019). “SelectiveNet: a deep neural network with an integrated reject option,” in Proceedings of the 36th International Conference on Machine Learning, 2151–2159. Available online at: https://proceedings.mlr.press/v97/geifman19a.html (Accessed August 13, 2025).

[B19] Géron A. (2022). Hands-on Machine Learning with Scikit-Learn, Keras, and TensorFlow. O'Reilly Media, Inc.

[B20] Grimmer J. Roberts M. E. Stewart B. M. (2022). Text as Data: A New Framework for Machine Learning and the Social Sciences. Princeton University Press. Available online at: https://go.exlibris.link/shrJy0z6 (Accessed August 11, 2025).

[B21] He H. Garcia E. A. (2009). Learning from imbalanced data. IEEE Trans. Knowl. Data Eng. 21, 1263–1284. doi: 10.1109/TKDE.2008.239

[B22] Hickman L. Thapa S. Tay L. Cao M. Srinivasan P. (2022). Text preprocessing for text mining in organizational research: review and recommendations. Organ. Res. Methods 25, 114–146. doi: 10.1177/1094428120971683

[B23] HMICFRS (2015). Increasingly Everyone's Business: A Progress Report on the Police Response to Domestic Abuse, 151. Available online at: https://assets-hmicfrs.justiceinspectorates.gov.uk/uploads/increasingly-everyones-business-domestic-abuse-progress-report.pdf (Accessed August 15, 2025).

[B24] HMICFRS (2019). The Police Response to Domestic Abuse: An Update Report, 58. Available online at: https://assets-hmicfrs.justiceinspectorates.gov.uk/uploads/the-police-response-to-domestic-abuse-an-update-report.pdf (Accessed August 7, 2025).

[B25] Home Office (2025). Crime Recording Rules for Front line Officers and Staff, 92. Available online at: https://assets.publishing.service.gov.uk/media/67ee9b2a199d1cd55b48c769/crime-recording-rules-for-frontline-officers-and-staff-2025_26-april-2025-update.pdf (Accessed August 5, 2025).

[B26] Karystianis G. Adily A. Schofield P. W. Greenberg D. Jorm L. Nenadic G. . (2019). Automated analysis of domestic violence police reports to explore abuse types and victim injuries: text mining study. J. Med. Internet Res. 21:e13067. doi: 10.2196/13067 30860490 PMC6434398

[B27] Kaufman S. Rosset S. Perlich C. Stitelman O. (2012). Leakage in data mining: formulation, detection, and avoidance. ACM Trans. Knowl. Discov. Data 6, 1–21. doi: 10.1145/2382577.2382579

[B28] Kohavi R. (1995). A study of cross-validation and bootstrap for accuracy estimation and model selection. Ijcai 14, 1137–1145.

[B29] Leitgöb H. Prandner D. Wolbring T. (2023). Editorial: Big data and machine learning in sociology. Front. Sociol. 8:1173155. doi: 10.3389/fsoc.2023.1173155 37229284 PMC10203698

[B30] Levenshtein V. (1966). Binary codes capable of correcting deletions, insertions, and reversals. Soviet Physics-Doklady 10, 707–710.

[B31] Long J. Harvey H. (2020). Femicide Census: Annual Report on UK Femicides 2018, 45. NIA. Available online at: https://femicidescensus.org/wp-content/uploads/2020/02/Femicide-Census-Report-on-2018-Femicides-.pdf (Accessed August 11, 2025).

[B32] Loshchilov I. Hutter F. (2019). Decoupled weight decay regularization. arXiv [Preprint] arXiv:1711.05101. doi: 10.48550/arXiv.1711.05101

[B33] Lum K. Isaac W. (2016). To predict and serve? Significance 13, 14–19. doi: 10.1111/j.1740-9713.2016.00960.x

[B34] Mikolov T. Yih W. Zweig G. (2013). “Linguistic regularities in continuous space word representations,” in Proceedings of the 2013 Conference of the North American Chapter of the Association for Computational Linguistics: Human Language Technologies, eds. L. Vanderwende, H. Daumé III, and K. Kirchhoff (Association for Computational Linguistics), 746–751. Available online at: https://aclanthology.org/N13-1090/ (Accessed August 14, 2025).

[B35] Molina M. Garip F. (2019). Machine learning for sociology. Annu. Rev. Sociol. 45, 27–45. doi: 10.1146/annurev-soc-073117-041106

[B36] Morris J. X. Lifland E. Yoo J. Y. Grigsby J. Jin D. Qi Y. (2020). TextAttack: a framework for adversarial attacks, data augmentation, and adversarial training in NLP. arXiv [Preprint] arXiv:2005.05909. doi: 10.18653/v1/2020.emnlp-demos.16

[B37] Myhill A. Hohl K. (2019). The “golden thread”: coercive control and risk assessment for domestic violence. J. Interpersonal Viol. 34, 4477–4497. doi: 10.1177/0886260516675464 27807208

[B38] Neubauer L. Straw I. Mariconti E. Tanczer L. M. (2023). A systematic literature review of the use of computational text analysis methods in intimate partner violence research. J. Fam. Viol. 38, 1205–1224. doi: 10.1007/s10896-023-00517-7 37358974 PMC10028783

[B39] Office for National Statistics (2024). Domestic Abuse Prevalence and Trends, England and Wales. Available online at: https://www.ons.gov.uk/peoplepopulationandcommunity/crimeandjustice/articles/domesticabuseprevalenceandtrendsenglandandwales/yearendingmarch2024 (Accessed August 15, 2025).

[B40] Pedregosa F. Varoquaux G. Gramfort A. Michel V. Thirion B. Grisel O. . (2011). Scikit-learn: machine learning in Python. J. Machine Learn. Res. 12, 2825–2830. doi: 10.5555/1953048.2078195

[B41] Phoenix J. (2023). Improving police data collection to measure repeat demand: a focus on domestic violence and abuse. Policing J. Policy Prac. 17:paad022. doi: 10.1093/police/paad022

[B42] Salton G. Buckley C. (1988). Term-weighting approaches in automatic text retrieval. Information Process. Manage. 24, 513–523. doi: 10.1016/0306-4573(88)90021-0

[B43] Sanh V. Debut L. Chaumond J. Wolf T. (2020). DistilBERT, a distilled version of BERT: smaller, faster, cheaper and lighter. arXiv [Preprint] arXiv:1910.01108. doi: 10.48550/arXiv.1910.01108

[B44] Song G. Ye Y. Du X. Huang X. Bie S. (2014). Short text classification: a survey. J. Multimed. 9, 635–643. doi: 10.4304/jmm.9.5.635-643

[B45] Turner E. Medina J. Brown G. (2019). Dashing hopes? The predictive accuracy of domestic abuse risk assessment by police. Br. J. Criminol. 59, 1013–1034. doi: 10.1093/bjc/azy074

[B46] Uysal A. K. Gunal S. (2014). The impact of preprocessing on text classification. Information Process. Manage. 50, 104–112. doi: 10.1016/j.ipm.2013.08.006

[B47] Victor B. G. Perron B. E. Sokol R. L. Fedina L. Ryan J. P. (2021). Automated identification of domestic violence in written child welfare records: leveraging text mining and machine learning to enhance social work research and evaluation. J. Soc. Social Work Res. 12, 631–655. doi: 10.1086/712734

[B48] Wang J. Wang Z. Zhang D. Yan J. (2017). “Combining knowledge with deep convolutional neural networks for short text classification,” in Proceedings of the Twenty-Sixth International Joint Conference on Artificial Intelligence ed, C. Sierra (Washington DC: Association for the Advancement of Artificial Intelligence), 2915–2921. doi: 10.24963/ijcai.2017/406

[B49] Wei J. Zou K. (2019). EDA: easy data augmentation techniques for boosting performance on text classification tasks. arXiv [Preprint] arXiv:1901.11196. doi: 10.18653/v1/D19-1670

[B50] Weir R. (2024). Differentiating risk: The association between relationship type and risk of repeat victimization of domestic abuse. Policing J. Policy Prac. 18, 1–12. doi: 10.1093/police/paae024

[B51] Whitehead S. (2024). The Quality of Police Recorded Crime Statistics for England and Wales (Systematic Review Programme). Office for Statistics Regulation, 1–53. Available online at: https://osr.statisticsauthority.gov.uk/wp-content/uploads/2024/05/OSR_police_recorded_crime_quality_review-1-1.pdf (Accessed August 7, 2025).

[B52] Wolf T. Debut L. Sanh V. Chaumond J. Delangue C. Moi A. . (2020). “Transformers: state-of-the-art natural language processing,” in Proceedings of the 2020 Conference on Empirical Methods in Natural Language Processing: System Demonstrations, eds. Q. Liu and D. Schlangen (Association for Computational Linguistics), 38–45. doi: 10.18653/v1/2020.emnlp-demos.6

[B53] Zhang D. Sensoy M. Makrehchi M. Taneva-Popova B. Gui L. He Y. (2023). “Uncertainty quantification for text classification,” in Proceedings of the 46th International ACM SIGIR Conference on Research and Development in Information Retrieval eds, H. Chen, W. E. Duh, H. Huang, M. P. Kato, J. Mothe, B. Poblete (New York, NY: Association for Computing Machinery), 3426–3429. doi: 10.1145/3539618.3594243

[B54] Zimmermann R. M. Allin S. Zhang L. (2023). “Common errors in machine learning projects: a second look,” in Proceedings of the 23rd Koli Calling International Conference on Computing Education Research ed, A. Mühling, I. Jormanainen (New York, NY: Association for Computing Machinery), 1–12. doi: 10.1145/3631802.3631808

